# Unmasking oral health stigma: a qualitative scoping review

**DOI:** 10.1186/s12903-025-07329-9

**Published:** 2025-12-17

**Authors:** J. Doughty, J. Booth, M. Smith, K. Saini, M. Paisi, A. Rodriguez, A. Levine, C. Bedos, V. Muirhead, C. Martins de Barros, C. Freeborn

**Affiliations:** 1 School of Dentistry, University of Liverpool, Liverpool, UK; 2 Peninsula Dental School, Faculty of Health, University of Plymouth, Plymouth, UK; 3 Bridgewater Community NHS Foundation Trust, Warrington, UK; 4 School and Nursing and Midwifery, University of Plymouth, Plymouth, UK; 5 School of Dentistry, University of Dundee, Dundee, UK; 6 Faculty of Dental Medicine and Oral Health Sciences, McGill University, Montreal, Canada; 7 Institute of Dentistry, Queen Mary University of London, London, UK; 8 Patient and Public Involvement Contributors, England, UK

**Keywords:** oral health, stigma, shame, dentistry

## Abstract

**Introduction:**

Health-related stigma can limit access to care, impair adherence to treatment, and negatively impact mental health and quality-of-life. Oral health stigma, defined as stigma arising from oral conditions that diverge from sociocultural norms, operates through labelling, stereotyping, othering, and exclusion. Oral health stigma can lead to shame, diminished self-confidence, and avoidance of dental care, creating a self-perpetuating cycle of poor oral health and reinforcing internalised and anticipated stigma. While previous research has explored the social implications of oral appearance, little is known about the broader concept of oral health stigma or strategies to mitigate it.

**Methods:**

This scoping review adopted Levac et al.’s six-stage framework. The review utilised data from qualitative studies to explore lived experiences of oral health stigma and consider ways to mitigate it. Patient and public involvement (PPI) informed the development of the research question, search strategy, and interpretation of findings.

**Results:**

Seventy-two qualitative studies were included, comprising 2,455 participants. Themes included stigma associated with physical appearance and attractiveness, judgement, labelling, and stereotyping. Consequences included low self-esteem, social exclusion, impacts to care seeking behaviours, and efforts to conceal oral appearance. Participants highlighted the transformative value of dental care and described coping strategies to build resilience. Other proposed solutions included fostering social connection and implementing trauma-informed, non-judgemental dental care.

**Conclusion:**

Oral health stigma has significant social and psychological consequences and impacts on care-seeking behaviours. Addressing it requires targeted interventions at multiple levels, including individual, community, professionals and wider system / policy.

**Supplementary Information:**

The online version contains supplementary material available at 10.1186/s12903-025-07329-9.

## Introduction

Health-related stigmas affect individuals by discouraging access to services, impairing adherence to treatment, diminishing mental health and social resources, and impacting quality of life [1–3]. Oral health stigma has been identified and defined as a unique health stigma harming people and groups with oral health that differs from the prevailing cultural norms (commonly held beliefs and expectations) [4]. Similar to other forms of health stigma, oral health stigma is the composite of labelling, stereotyping, othering, and social exclusion [5].

The appearance of the mouth and the teeth can impact how people feel about themselves and how they experience themselves in relation to the world around them. In the global West, there is a general understanding that straight white teeth are aligned with success, while visible signs of dental disease or differences can attract stigma and discrimination [6–8]. For example, oral appearance can lead to bullying, perceptions of lower intelligence, impact relationships and even affect employment opportunities [8, 9]. Individuals may feel embarrassed or ashamed about their oral health, have low self-confidence, and avoid socialising [9, 10]. Oral health stigma impacts self-confidence through psychological impacts, aesthetic concerns and social sensitivity, triggering shame tied to basic expressive functions like smiling and speaking [11, 12].

Oral health stigma can lead to healthcare avoidance, negatively impacting oral health outcomes [13]. For example, embarrassment, more than fear of pain, has been implicated in long term dental avoidance and mouth-hiding behaviours [14]. This relationship can be cyclical and self-driving, whereby poor oral health and anticipated or enacted stigma generates fear of stigma from healthcare professionals, which in turn leads to healthcare avoidance, thus negatively impacting oral health status.

Studies have highlighted that oral health stigma operates at the intersection of oral health, identity and cultural context [11, 15]. Factors including gender, age, ethnicity, measures of socioeconomic status, cultural norms including media, power and privilege collectively influence perceptions and experiences of oral health stigma and discrimination [4, 7, 11, 15–17].

The concept of oral health stigma has predominantly been addressed by 1) uni-dimensional measures of stigma such as embarrassment or self-consciousness or employment, 2) by focusing on singular oral and dental conditions or 3) at the intersection of oral health and stigmatised identities, such as homeless people or indigenous populations. Oral health stigma arises from any differences in oral health and may manifest in multiple ways, including shame, avoidance of dental care, and self-medication [4, 18]. Therefore, theoretical frameworks are required that capture oral health stigma beyond the constraints of a singular manifestation, condition or characteristic.

Although research addressing the impact of oral appearance has been conducted, there is a paucity of research explicitly exploring the broader concept of oral health stigma or the destigmatisation of different oral health statuses [4]. Therefore, this scoping review aims to explore the understanding, experiences and consequences of oral health stigma and identify suggestions to address it.

### Research question

This review focused on three research questions:

1. How is oral health stigma understood and experienced in the published qualitative research literature?
2. What are the consequences of oral health stigma?
3. What strategies have been adopted to address oral health stigma?

## Methods

### Scoping review methodology

Scoping reviews are grounded in a transparent methodological process and “move beyond description of a body of evidence to derive new insights through integration and/or critique” [19]. The method is appropriate to clarify and identify key characteristics and concepts related to oral health stigma [20]. The Levac et al. [21] scoping review methodology was used to ensure the rigour of the scoping review process. The 5-stage scoping review process included (1) identifying the research question, (2) identifying relevant studies, (3) study selection, (4) charting the data, (5) collating, summarising and reporting the results, (6) consultation. The scoping review was limited to the inclusion of qualitative data, drawing on it’s epistemological strengths to explore the insider perspective and lived experience of people situated within their cultural context.

### Human ethics and consent to participate

This study utilised published materials and did not actively recruit participants; therefore no consent was required to undertake the review. The patients and public members involved in the study acted as co-researchers, not participants, and therefore formal informed consent was not required. Human Ethics and Consent to Participate declarations were not applicable. The study included only peer-reviewed primary studies.

### Patient and public involvement (PPI)

PPI with scoping reviews can improve relevance, capture a broader range of results, and in the case of this scoping review, it offered members of the public an opportunity to share their lived experience of oral health stigma in a safe space as part of their own healing journey [22]. The scoping review integrated PPI at the following stages: establishing the value of the research question, developing the literature search strategy, interpretation and presentation of results and co-authorship (Supplemental material 1). Patients and the public with lived-experience of oral health stigma were identified from the People In Research Website. People who responded to the study advert were invited to complete an informal survey of oral health perceptions and stereotypes. Responses were obtained from 28 people who self-identified as experiencing oral health stigma (PPI survey tool supplemental Fig. 1) Following the survey, a discussion about oral health stigma was held with three members of the public with self-identified lived experience of oral health stigma. The PPI contributors included one male and two females. Ethnicities included White British, Black British and Mixed ethnicity and spanned different age groups. Patients and the public believed that the topic of oral health stigma was essential and timely and suggested that we also focus on ways to understand how people have overcome oral health stigma. We also established that the public had an inherent understanding of what is meant by the term oral health stigma. Patients and the public with lived experience of oral health stigma reviewed the study findings and offered their own interpretations, reflections and understanding. Involving patients and the public revealed several important and real-world concepts; for example, the efforts to hide oral appearance by using filters on phones. PPI also highlighted that the experience of oral health stigma is not universal, while some people are affected by social media, others do not perceive it has an impact on oral health stigma. Two members of the public have reviewed the findings, contributed to their interpretation and co-authored the manuscript.

### Inclusion and exclusion criteria

The Doughty et al. definition of oral health stigma and PPI guided our understanding of inclusion criteria for oral health stigma [4]. Only qualitative studies were included in the review to draw on the epistemological strengths of qualitative research to explore the insider perspective and lived experience of people situated within their cultural context. No primary qualitative studies were excluded from this review if they provided insight into the research questions. Consistent with the scoping review methodology, this scoping review does not intend to identify gaps where the research is itself of poor quality since quality assessment does not form part of the scoping review remit [23].

The date range of the literature spanned from January 2014—July 2024 to ensure that the articles reflected the contemporary societal contexts including, for example, online and media stigma. Only studies published in English were included in the review. Studies undertaken in any country were eligible for inclusion. The SPIDER (Sample, Phenomenon of Interest, Design, Evaluation and Research) tool is designed to supported the identification of relevant qualitative studies and can be used in preference to the PICO tool when undertaking qualitative reviews (Table 1) [24].

**Table 1:** Inclusion and exclusion criteria structured using the SPIDER tool

SPIDER	Inclusion	Exclusion
Sample	Dental or other healthcare professionals, members of the public irrespective of age, gender, ethnicity, or other characteristics. The sample must have described lived-experience of the phenomenon of interest	
Phenomenon of Interest	Typologies of stigma as applied to oral health, including discrimination, prejudice, judging, shaming, blaming, self-esteem, embarrassment, social isolation, labelling, stereotyping and other presentations of stigma, public perspectives and experiences, being treated differently. Oral health stigma may be experienced as self-stigma, stigmatising others, institutional/professional stigma, public stigma or secondary stigma	Stigma related explicitly to a non-dental stigmatised characteristic, e.g. HIV or homelessness or overweight or mental illness that does not relate or intertwine with experiences of oral health stigma (i.e. is not intersectional stigma)
Design	Interviews, focus groups, yarning circles and other qualitative methods including creative methods such as video or imagery. Narrative or thematic analysis	Quantitative methodologies e.g., Cross-sectional surveys, Cohort studies, case–control studies, expert opinions, and editorial commentary. Literature reviews (used only for reference searching). Content analysis where qualitative data has been quantified
Evaluation	Experiences and perceptions	Any other means of evaluation
Research type	Qualitative studies and qualitative components of mixed methods studies	Quantitative studies and quantitative components of mixed methods studies

### Search strategy

The search strategy encompassed scoping review framework stage 1. The strategy was informed by the core research team, which included six academics with an interest in the field of oral health and stigma and with patients and the public who helped to identify key lay terms around oral health stigma. Further, JD and VM have previously authored a narrative review of oral health stigma and had preliminary knowledge of the literature [4]. The working definition of oral health used for this synthesis appeals to that described by the World Dental Federation as related to: “the ability to speak, smile, smell, taste, touch, chew, swallow and convey a range of emotions through facial expressions with confidence and without pain, discomfort and disease of the craniofacial complex” [25]. A pilot search was undertaken to establish the reach and comprehensiveness of the search terms related to 1) shame and stigma terms 2) dentists and oral health terms 3) qualitative methodological terms. The findings of the pilot search supported the further development and refinement of the search strategy in conjunction with PPI development (Supplemental Table 2). Relevant papers identified in the pilot search were also included in the scoping review. The search strategy was enacted on Web of Science and Medline (via Ovid) on the 16th July 2024. Search terms were applied to titles and abstracts.

### Data extraction and management

Data extraction and management encompassed scoping review framework stages 2 and 3. Endnote was used to facilitate deduplication. Rayyan was used for the title and abstract screening process, which was undertaken by JD, JB and MS [26]. Rayyan enabled the researchers to determine whether studies were to be included, excluded, or require discussion. The Notion app was used for the full-text review and data extraction (JD, JB, MS and KS). Two reviewers assessed each full-text paper for inclusion and data extraction (JD and JB, MS or KS). Disagreements were resolved through discussion and a third reviewer consulted where required. The data extraction form included authors, date of publication, journal title, population, participants, phenomenon of interest, design, evaluation, overview/summary of paper, reason for exclusion and illustrative quotes from the papers. Additionally, a column was labelled for each deductive coding stratum to identify which type(s) of stigma were addressed by each article. The data extraction form was piloted by JD and KS on ten cases.

### Data analysis, synthesis and interpretation

Data analysis and synthesis includes stages 4, 5 and 6 of the scoping review framework. Extracting and synthesising the data was undertaken by four researchers (JD, KS, JB and MS). Data were extracted into a charting form. The form was iteratively developed with an initial pilot followed by discussion and adaptation. Data were collected about the study details from the methods sections . Data from the results sections including interpretations and quotes were extracted. Additionally, binary data were extracted for common presentations of stigma as described by Stangl et al. in their cross cutting Health and Stigma Discrimination Framework enabling a numerical assessment of stigma typologies [27] Data were collated and presented as numerical summary and a thematic analysis [28].

The principles of Thomas and Harden’s [29] thematic synthesis of qualitative research guided the analysis and interpretation process. All relevant text from results sections of the included studies and relevant quotations were organised into the scoping review charting framework which allowed for comparisons across the data sources. Initially descriptive themes were generated that related to different known types of stigma closely reflecting the study data. Thereafter, the charted data and descriptive themes underwent an interpretation stage to generate novel interpretations from the findings. The interpretation process was undertaken with research experts in the area of stigma, social science and PPI contributors (VM, JD, CB, AD, AR, CMB, CF); therefore, “*These interpretations will not be found in any one research report but, rather, are inferences derived from taking all of the reports in a sample as a whole"* [30]*.* The overall findings from the sample were integrated into a conceptual framework of oral health stigma agreed with all authors and PPI contributors.

### Findings

**Fig. 1:**
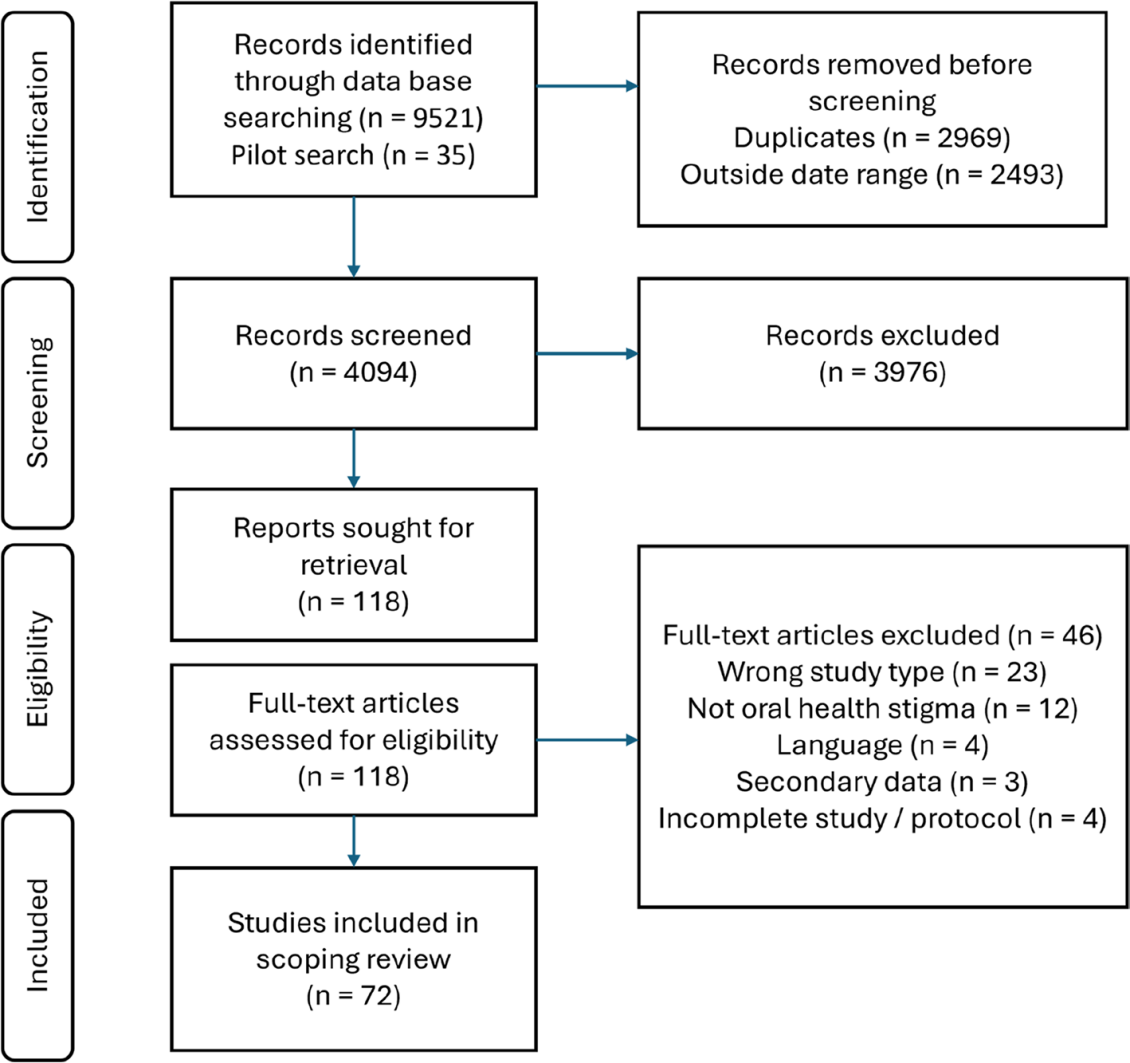
PRISMA flow diagram of studies included in the scoping review

**Fig. 2:**
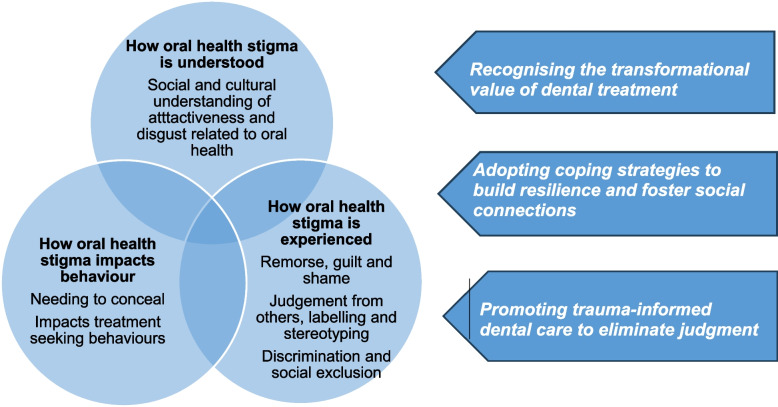
Concept map illustrating understanding experiences and consequences of oral health stigma and approaches to address it

Of the 9521 papers that were initially identified, 72 papers were included in the review (Fig. 1). Across the 10-year period, there was no clear trend in number of publications; however, there were peaks in 2019 (*n* = 12), 2021 (*n* = 13) and 2022 (*n* = 11) (Fig. 2).

### Participants

There were 2,455 participants across 72 studies; the number of participants in the studies ranged from 6 to 226 [31, 32]. The participants varied in ages from seven to 87 years old [33, 34] Most studies involved perspectives of adults either about themselves or on behalf of their children or their patients/clients [16, 18, 31–33, 35–85]; fewer studies involved children/adolescent participants [34, 49, 64, 86–94]. Across the studies, participants were recruited either because they represented the general population of adults and children, had or were related to someone with a specific oral condition or specific treatment, or had a particular characteristic, health condition or phenomenon of interest, or were a member of health, social or dental care staff (Details and references in Tables 2 and 3).

**Table 2:** Sample characteristics

Sample group	Sample characteristic	References
General population (no focus on specific health or oral characteristic)	Older adults	[40, 53, 76]
	Adult	[40, 55, 56, 83, 84]
	Child/adolescent	[34, 92, 93]
	Parent/caregiver	[49, 57]
Health, social or dental professional	Dental professionals	[79, 83, 85, 89, 95–97]
	Other heathcare staff	[79, 89, 98]
	Social support staff	[68, 98, 99]
	Teaching staff	[85]
Oral condition	Amelogensis or dentinogenesis imperfecta	[47, 48, 86]
	Ectodermal dysplasia or Oligodontia	[42, 46]
	Cleft lip and palate	[50, 65, 89]
	Oral cancer	[61]
	Facial disfiguration	[36]
	Early childhood caries	[57]
	Tooth loss (deciduous)	[77]
	Tooth loss (permanent)	[80]
	Malocclusion	[87, 90, 94]
	Noma	[69]
Dental treatment or approach	Implants	[45]
	Orthodontics	[52, 63, 94]
	Orthagnathioc surgery	[35, 59, 88]
	Periodontics	[51]
	Dentures or obturators	[38, 41, 46]
	Sodium Diamine Fluoride	[51, 64]
	Humanising dentistry	[96]
	Diet diary	[49]
	Root canal treatment	[71]
Characteristic of interest	Indigenous population	[16, 32, 33, 74, 75]
	Homeless people	[44, 72, 73]
	Irregular dental attendance	[60, 81, 82, 96]
	Dental aid schemes and financial challenges	[81, 97, 100]
	People in prison	[68, 98]
	Hospitalisation	[70]
Health condition	Substance use (opioid maintenance therapy, alcohol)	[62, 67, 78]
	Mental ill health	[39, 66, 67, 79]
	Dental anxiety (history of trauma)	[54]
	Rheumatoid arthritis	[37]
	Eating disorder	[43]
Phenomenon	Oral health stigma	[18]

### Type of study

Both qualitative and mixed methods studies were included in the review; however, only the qualitative components of the mixed methods study were utilised. There were 54 studies that used interviews [16, 18, 31, 32, 35–38, 40–43, 46, 47, 49–55, 58–67, 69, 70, 72, 73, 76, 77, 79–82, 84, 86–90, 92, 94–98, 101] as their qualitative method, nine undertook focus groups [34, 45, 48, 57, 68, 71, 85, 91, 93], and nine used both interviews and focus groups [33, 39, 44, 56, 74, 75, 78, 83, 99]. In addition to interviews, four studies also included qualitative documentary analysis i.e., diaries [49, 51, 87, 97].

### Country of publication

Studies were published from across the globe studies in Europe (*n* = 33) [35, 40–42, 44, 47–49, 53–56, 60–64, 69, 70, 72, 73, 78, 82–86, 88–90, 95, 98, 101], Australasia (*n* = 11) [16, 32, 33, 39, 43, 51, 74, 75, 87, 94, 97], North America (*n* = 13) [31, 34, 37, 52, 58, 66–68, 79, 81, 92, 96, 99], South America (*n* = 8) [36, 38, 45, 57, 59, 76, 77, 80], Asia (*n* = 5) [18, 46, 71, 91, 93] and Africa [50, 65] (*n* = 2) .

### [Types of oral health stigma

Aspects of oral health stigma (i.e. drivers, outcomes, reduction) and typologies of oral health stigma (i.e. self-stigma, secondary stigma) were charted in the scoping review framework. More than two thirds of studies related to the following aspects of stigma: drivers of stigma (*n* = 45, 63%), anticipatory stigma (*n* = 48, 67%), self-stigma (*n* = 45, 63%), shame (*n* = 49, 68%) and health and social impacts (*n* = 48, 67%). Fewer studies explored intersectional stigma (*n* = 25, 35%), secondary stigma (*n* = 18, 25%) and discrimination (*n* = 27, 38%) and approaches to address stigma (*n* = 37, 51%). Table 3 presents a summary of the topics identified within the qualitative literature most studies covered multiple types of stigma.

**Table 3:** Mapping of typologies of oral health stigma

Oral health stigma aspect	n (%)	References
Drivers	**45 (63)**	[18, 32–34, 37–39, 43, 44, 46, 48, 50–52, 54, 57–60, 62, 63, 65, 67, 69, 72–75, 78, 79, 81–84, 87–89, 91–94, 96, 98–100]
Intersectional stigma	**25 (35)**	[16, 18, 32, 37, 39, 43, 44, 50, 58, 62, 67, 72, 74, 75, 78, 79, 82, 83, 87, 91, 92, 94, 96, 98, 100]
Anticipatory stigma	**48 (67)**	[16, 18, 32–36, 39, 41, 42, 44, 45, 48–52, 57–64, 67, 69, 70, 72–76, 79, 81, 82, 84, 86, 87, 89, 91–94, 98–101]
Self-stigma	**45 (63)**	[16, 18, 32, 33, 35–39, 41, 42, 44, 45, 48, 49, 51, 52, 54, 58–62, 66, 67, 69, 72, 73, 75, 76, 78, 79, 81, 82, 86–94, 100]
Secondary stigma	**18 (25)**	[16, 32, 33, 40, 49, 50, 57, 62, 64, 65, 67, 70, 74, 80, 85, 90, 99, 101]
Shame	**49 (68)**	[16, 18, 33–39, 41, 42, 44, 46, 49, 51, 52, 54, 58–62, 66, 67, 69, 72–76, 78–82, 84–94, 98–100]
Discrimination	**27 (38)**	[16, 18, 36, 39, 48, 52, 57, 58, 60–62, 65, 67, 69, 72, 74, 78, 80, 81, 85–87, 93, 96, 98–100]
Health and social impacts	**48 (67)**	[16, 18, 33, 34, 36, 38, 39, 41, 42, 44, 48, 49, 51, 52, 54, 55, 58–61, 63, 65, 72, 74–76, 78–84, 86–91, 93, 94, 96, 98–100]
Approaches to reduce stigma	**37 (51)**	[16, 33–36, 40, 42–46, 48, 51, 52, 54, 58, 59, 63, 69, 72, 75, 76, 79, 82–84, 86–92, 94, 98–100]

### Themes: Intersecting factors to explain oral health stigma

Themes were identified from the included studies on oral health stigma that gave insight into the understanding, experiences and consequences of oral health stigma and ways to address it and are illustrated in a concept map (Fig. 2) and fully described below. Example quotes supporting each theme and subtheme are presented in Supplemental Table 3.

### Oral health stigma: understood in physical terms related to disgust and attractiveness

Many participants primarily described and understood stigma as a physical attribute inextricably linked to how other people perceived their oral health status; this was linked to signs and symptoms that could be observed or detected by others and was linked to ideas around attractiveness and disgust [43, 52, 55, 63, 90]. Females with experience of eating disorders recognised that this potent desire to have attractive teeth had increased over time leading to "*a hyper-focused awareness on beauty*” [43]. Children and adolescents prioritised attractiveness and social acceptance as stronger motivators to have “*pearly white teeth*” than for health and prevention purposes [34, 92]. Young people with malocclusion felt that teeth were "*one of the most stand out features of your face*” and people “*judge you when they first meet you*” because of the appearance and function of your mouth and teeth [90]. Orthodontic patients felt that oral health stigma was perpetuated in the widespread and pervasive commercial media and celebrity culture influencing societal norms about attractiveness and that there was a fine line between what was considered abnormal requiring treatment and what was a variant of normal [52]. Participants from Brazil who completed orthodontic and orthognathic surgery for Class II or Class III malocclusions described their motivation for treatment was driven by their appearance.

Participants with conditions such as noma, periodontal disease or who wore obturators understood that some oral conditions could provoke disgust. Some people with periodontal disease worried about people smelling their bad breath [51]. Obturators led people to fear that cleaning the prosthesis after eating “*would turn people’s stomach*” [41]. People with Noma understood that people in their community found them disgusting and they described finding themselves disgusting [69]. The appearance of amelogenesis imperfecta made people feel that their teeth looked like they hadn’t been brushed or had poor oral hygiene which made children feel “*unhealthy and well disgusting*” [86].

### Oral health stigma: experienced as judgement, shame and exclusion

Oral health stigma was vividly described in accounts of regret, self-blame and stereotypes or labels related to people with oral health differences. For example, people with periodontal disease and rheumatoid arthritis described their internalised sense of shame, deep remorse, guilt, and self-blame for oral conditions perceived as being preventable [37, 51] Several studies [16, 47–49] also explored stigma experienced by parents who felt guilty about their children’s oral health. Indigenous parents of children with visible decay often experienced embarrassment, perceiving this as a reflection of neglect. Parents of young children explained their fear, guilt, vulnerability, and humiliation about their children’s oral health and blamed themselves for inherited conditions such as amelogenesis imperfecta [86]. Older participants were ashamed about their poor dental appearance seen which was experienced as as pitiful and a sign of ageing [53]. In African cultures, shame was compounded by cultural myths and misinformation about the infectivity of oral conditions such as Noma believe to be a sign of evil [65, 69]. Participants with orthodontic concerns felt that having a protrusive lower jaw gave an impression of a person who lacked intelligence or vitality. Brown staining, dental decay, oral cancer, or missing teeth were seen as signs of self-neglect, self-sabotage; or behaviours typically seen in drug or alcohol abuse, poor hygiene, laziness or people with low social status/value [39, 40, 48, 51, 52, 61].

Participants portrayed their experience of discrimination, encountering bullying, teasing, staring, name-calling, stigmatising questions, assumptions about hygiene practices, avoidance, pity, expressions of disgust and unkind comments [42, 47, 48, 57, 63, 68, 69, 77, 89, 91]. They faced wider societal discrimination related to employability, struggling to reintegrate into society after transition from the justice system because of the poor dental appearance and feeling social ostracized from communities [57, 63, 86, 89, 91, 93].

Participants had also experienced discrimination in professional settings through their interactions with dental professionals, who were often seen as disapproving or condescending authority figures who humiliated people, made incorrect assumptions, and were “*always telling you what is wrong*” but failing to acknowledge what people were doing well [49, 51, 84, 85]. Discriminatory dental care was perceived as related to racism and a legacy of colonialism, the structure of dental payment systems (e.g. Medicaid), and intersecting stigmatised identities (e.g. mental ill health, homelessness, Indigenous peoples) and behaviours (e.g. drug addiction, alcoholism) [62, 67, 74, 78].

### Oral health stigma: Self-consciousness driving concealment behaviours

Differences in oral health that might be noticed by others caused self-consciousness, which impacted self-esteem and -confidence [44, 56]. People with dental decay, amelogenesis or dentinogenesis imperfecta, malocclusions, missing teeth, periodontal disease or who wore obturators made efforts to conceal their mouth and teeth. Specifically, this was linked to the risk of exposing the dental issue of concern and the potential to be judged or thought about in a negative light. Females were reported to experience more embarrassment and self-consciousness related to differences in dental appearance than males [32, 94]. Participants with any visible oral differences described covering the mouth with lips or a hand, not smiling in photographs, pretending to scratch their nose or avoiding getting close to others [32, 35, 37, 41, 51, 69, 72, 73, 77, 80, 81]. Some people avoided socialising or meeting new people, tolerated painful prostheses, and avoided talking about their oral condition [32, 35, 37, 41, 51, 69, 72, 73, 77, 80, 81]. Masking methods for bad breath included mints or chewing gum. Patients avoided treatments like Silver Diamine Fluoride (SDF) due to fears of negative perceptions related to the treatment’s side effect of staining teeth [64, 101]:

Some people with dental conditions related to self-neglect and poor oral hygiene avoided treatment seeking, concealed important information, felt anxious about attending the dentist and failed to adhere to practices such as diet diaries [18, 51]. Contrastingly, participants with congenital conditions such as amelo- or dentinogenesis imperfecta, malocclusions, or cleft lip and palate actively sought treatment to address their dental issues.

Some participants avoided dental care, anticipating and experiencing feeling judged about their oral health or oral hygiene in their interactions with dental professionals [52]. Specifically, this led Indigenous populations and people who use drugs or had mental health conditions to describe how these characteristics combined with poor oral health led dentists to assume that they were neglecting their oral health and oral hygiene and had poor attendance patterns.

As a result of avoiding dental care, participants described entering into a cycle of accumulated dental disease, embarrassment and shame about their deteriorating oral health paradoxically increasing their desire to avoid the dentist [60]. Feelings of shame about oral health "*layered like an onion*", taking primacy over barriers such as resources and the perceived importance of oral health in a web of causation that discouraged people from accessing dental services [60, 82].

### Addressing oral health stigma

Three key means of addressing oral health stigma were derived from the literature, these centred around addressing oral health issues with treatment, building resilience and social connections, and dental services actively engaging in trauma informed dental care. No population level oral health stigma policies or interventions were identified in this study. The data underpinning each approach are detailed below.

### Recognising the transformational value of dental treatment

Participants described using treatment as the first-line approach to restore aesthetics and alleviate dental disease. For example, improved dental appearance, as opposed to function, was the focus for orthodontic patients [63]. Dental treatment enabled homeless people and people with tooth loss to “*get back to normal… to that person you were before*” and resume employment or create social relationships with a transformative power to affect confidence and quality of life [38, 44, 72]. People undergoing orthodontic treatment shared how treatment improved their self-image, which was intricately linked to their self-confidence and social interactions [63, 94]. For people experiencing addiction, or incarceration, restoring oral health was a means to reconstruct self-image and self-esteem, key factors of reintegration [98]. People with periodontal disease recalled how dental treatment reduced their concealment behaviours; people smiled more, looked at their teeth in the mirror, resumed social activities and reduced chewing gum to hide halitosis [51]. Treatments that people generally felt improved their experiences of oral health stigma included correcting malocclusions, surgical closure of Noma wounds, providing crowns to disguise amelogenesis imperfecta, or dentures and implants to replace missing teeth [38, 45, 46, 69, 88]. However, participants who had disfigurement or obturator after surgery for oral cancer, recession from periodontal or endodontic surgery, visible staining following Silver Diamine Fluoride application or who felt aged by dentures experienced a new stigma after treatment [41, 51, 64, 71, 101].

### Adopting coping strategies to build resilience and foster social connections

Participants also described how they used individual, personally meaningful coping strategies to foster resilience to alleviate stigma. For example, some participants with post-cancer facial disfigurement created nuanced coping mechanisms to reconcile with their altered appearance. For example by deepening religious beliefs, joining social support networks, engaging in acts of resilience like reconstructing their self-image or reframing disfigurement as a symbol of survival and strength; a personal narrative of having “*beaten*” the disease [36]. However, others accepted or resigned themselves to changes [36]. Another coping strategy mentioned by people with lived experience of oral health stigma from Pakistan involved fostering social connections with friends who understood and were empathetic to their struggles [18]. Beyond intimate connections, patients and dental consultants described the role of education and awareness, including campaigning within the communities to demystify oral health issues, reduce oral health discrimination by accepting differences in oral health and encourage people to seek support from dental professionals regarding oral health issues [18]. Dental consultants also raised concerns that training in oral health stigma was insufficient in dental education and advocated for workshops and guidelines in managing stigma in dental practice.

### Promoting trauma-informed dental care to eliminate judgment

Studies described using systemic care approaches and trauma-informed dental services to provide non-judgmental, positive interactions with healthcare providers. This alleviated dental anxiety, reduced shame, and encouraged ongoing engagement with dental care, particularly for people with a history of trauma [54]. People with lived experience of oral health stigma believed that dental professionals can help to minimise shame by using empathetic listening, validating, being friendly and reassuring and balancing information to include positive observations [18]. Dental patients and parents felt that collaboratively looking at issues rather than alienating, blaming or lecturing with criticism was a helpful approach [83], [85]. Indigenous people felt that “*removing shame and judgement from a clinical relationship and environment is crucial in building trust between patient and practitioner*” [33, 84]. 

### Concept map

Figure 2 presents a summary concept map that brings together the key ideas about how oral health stigma is understood, experienced and subsequently manifested as behaviours. The concept map highlights recommendations for addressing oral health stigma that appeared in the literature. The circles in the diagram are interwoven, highlighting how people first must understand that their oral health is different from that considered normal or acceptable within their cultural context. They experience this difference in relation to its social consequences through commonly understood stereotypes or labels that are a source of judgment, shame and exclusion whether real or imagined. To avoid negative experiences, people conceal the differences in their mouth and teeth from the public and dental professionals.

## Discussion

### Statement of principle findings

This scoping review aimed to map and synthesise the current qualitative literature surrounding the concept of oral health stigma, whilst previous research has explored the social implications of oral appearance, little is known about the broader concept of oral health stigma or strategies to mitigate it. This scoping review highlighted that oral health stigma is a ubiquitous phenomenon that affects people of all ages from childhood to older age, crosscuts oral health conditions and treatments, intersects with pre-existing stigmatised identities and presents across multiple types of stigma. However, only one study explored oral health stigma as a phenomenon outwith of specific oral conditions or stigmatised identities.

The findings from the study produced a concept map (Fig. 2) that described oral health stigma as 1) being understood in relation to physical terms related to attractiveness, 2) being experienced as remorse, guilt, shame and judgement, discrimination and social exclusion and 3) impacting on self-esteem and self-confidence leading participants to hide their oral condition and affecting treatment seeking behaviours. The diagram illustrates how these three concepts are interconnected and can overlap, compounding an individual’s experience and consequence of oral health stigma. For example, someone may be worried about others being disgusted by their oral appearance, as a result, they may feel judged or treated differently, which negatively impacts their self-esteem. To avoid these negative experiences around other people, they may avoid socialising or avoid smiling and talking.

People who had visible dental disease related to self-care neglect described feeling regret and shame for their oral condition and some avoided dental services or self-medicated [18]. Contrastingly, people with congenital conditions such as amelo- or dentinogenesis imperfecta or cleft lip and palate actively sought treatment to address their dental issues. This difference in treatment-seeking behaviours may be explained by societal norms around oral health. In the stigma literature, there have been mixed findings suggesting that stigma can both impede and encourage treatment adherence for people with conditions including HIV or TB [102].

Oral health stigma combines the features of other stigmatised conditions such as the behavioural symptoms of mental illness, as well as the perceived preventability of conditions linked to immorality, such as smoking and lung cancer, and conditions of infectivity such as leprosy [3, 103, 104]. Oral health, therefore, is unique in the multiple understandings of the stigma it ignites. For example, people with hygiene-related oral conditions, experienced stereotypes as related to poor self-care (immorality), laziness or poverty (negative character traits), and disgust (contagion avoidance) [105]. In the Global West, oral hygiene is a societal norm. Studies have highlighted that people who violate hygiene norms are consistently stigmatised. Hygiene violations are closely linked to ideas around morality as opposed to disease/contagion and hygiene violators are shown little empathy [106]. In the wider literature, empathy has been reported as key to increasing patient compliance with, and adherence to, treatment, greater patient satisfaction and reduced dental anxiety [107].

This scoping review presented how participants perceived dental professionals attitudes as judging, criticising or blaming. Stigma in healthcare settings undermines diagnosis, treatment and successful health outcomes [108]. Patients value a caring dentist who respects them, listens to their concerns and doesn’t blame them for their oral health status [109]. Participants in this scoping review identified core values that supported dental care, including validating, reassuring and giving positive observations about their oral health behaviours. Dolezal and Gibson argue that shame-sensitivity is essential for a trauma-informed approach to healthcare. The three central concepts of shame-sensitivity include: acknowledging shame, avoiding shaming, and addressing shame for the individual by building shame-resilience and prioritising relationships based on dignity, respect, empathy and trust [110]. Shame and stigma are distinct yet inter-related concepts and these principals could be applied for anyone experiencing oral health stigma.

Of the approaches to addressing oral health stigma, the first line approach across many studies was to seek transformational dental care to alter appearance or health status, thereby removing the source of oral health stigma. Some oral conditions may not have a definitive cure or be resolved by treatment. For these conditions oral health stigma was addressed by adopting coping strategies to build resilience, fostering social connections and the wider community, and promoting trauma-informed dental care to eliminate judgment [18]. Very few strategies to address oral health stigma were identified in this scoping review and non related to population-level public health interventions. Interventions to address healthcare stigma have been criticised for failing to target multiple levels of staff or ecological levels within healthcare facilities or leverage interactive technology [108].

Anti-stigma training and awareness public campaigns can be an avenue to improve knowledge, attitudes, communication skills and perceived empathy [111]. Multi-level approaches have been lauded as the most appropriate means to tackle health-related stigmas [112]. Stigma interventions can be targeted at 1) individuals to enhance coping strategies or change attitudes and behaviours toward stigmatised people. 2) dyadic or small group interactions, 3) socio-political approaches including laws and policies. Such interventions would benefit from longitudinal follow-up across systems. Interventions between levels reciprocally affect one another [112]. Therefore, any intervention toward the amelioration of oral health stigma would benefit from a multi-level focus for maximum benefit.

### Gaps in the literature

In this scoping review we identified sparse literature discussing individual, community and professional opportunities for interventions. No population level oral health stigma policies or guidelines were described in the included studies. Most studies related oral health stigma to a specific condition or characteristic, only one study explored oral health stigma as a specific phenomenon of interest in its own right. Additionally, most studies were conducted in high-income countries in the Global West; therefore, we cannot make assumptions about the nature of oral health stigma in low and middle-income countries (LMIC). Stigma may be more severe or differently expressed in LMIC, and the absence of research is a gap of global health significance. Few studies reported secondary stigma and these were predominantly linked to the perspectives of parents; further studies would benefit from exploring the impact of secondary oral health stigma related to spouses or caring responsibilities for adults. Further research is required to understand the experiences and impact of oral health stigma in these countries.

### Recommendations

#### Research

Further research is needed to explore the potential benefits of community engagement via platforms such as social media to promote patient advocacy and reduce oral health stigma and discrimination. Research is needed to understand how people may benefit from participation in engaging with support groups with other people experiencing oral health stigma and to understand what approaches can enhance personal resilience. At a population level, there may be value in understanding what approaches can change cultural perspectives around oral health for example by launching media campaigns around acceptance of oral health differences and stereotypes.

#### Clinical practice

Dental professionals would benefit from embedded education about the experiences and impacts of oral health stigma and stigma/shame-sensitive dental care in their undergraduate curricula. Implicit bias training could support dental professionals to recognise and address their own potential biases toward patients with oral health issues. Interdisciplinary learning with other healthcare professionals could support the understanding of the universality of stigma across other healthcare conditions and the impact of intersectional stigma. Practice resources could include conversation guides to help dental professionals integrated advocacy into their daily practice or peer mentoring programmes where experienced dentists have the opportunity to share strategies for trauma-informed care. More specific training on the definition, causes, and experiences of oral health stigma may have the potential to create sensitive and well-equipped dentists who have improved interactions with patients experiencing oral health and multiple stigmas.

#### Community

Funding for community-based oral health advocacy programs could engage patients directly, without the additional barrier of having to access dental services to receive support. Additionally, dental professionals should be able to signpost patients to local oral health advocacy groups or support and encourage them to organise their own community-based groups. Dental professionals could advise patients on approaches to building resilience and leaning in to existing relationships that can support them to navigate their experiences of oral health stigma and to engage with treatment seeking where appropriate.

#### Policy

Policy or guidance documents focused on understanding and addressing oral health stigma are crucial to supporting people to access dental services and to cope with the social implications of having differences in oral health. These policies would support the dental profession to understand the profound implications of oral health stigma for mental wellbeing and the social integration of their patients. Policy reforms should integrate oral health stigma into broader public health and mental health strategies for cross-sector collaboration. Partnerships between policymakers, professionals and patient advocacy groups could co-develop policy and guidelines to address oral health stigma at all levels.

### Strengths and limitations

This is the first study to explore the body of qualitative literature on oral health stigma. The study does not include quantitative literature; therefore, it does not cover the full spectrum of dental research that may contribute to the understanding and conceptualisation of oral health stigma. Most studies in this review were authored in Europe, North and South America, and Australia. There were few studies undertaken in Africa or Asia and this could have implications for how oral health stigma is perceived on a global level and how it is understood in relation to cultural or geopolitical differences outside of the Global West. Few studies addressed oral health stigma beyond treatment seeking, therefore the recommendations for other approaches to address oral health stigma are weak and may not be generalisable. Qualitative research poses challenges for generalisability; therefore, the findings and recommendations must be considered relative to the population or oral condition under study and the cultural context. Consistent with the scoping review methodology, this review did not undertake quality assessment and therefore it cannot identify research gaps where the research itself is of poor quality.

## Conclusions

This scoping review highlights that oral health stigma is understood primarily in relation to physical appearance, attractiveness, and perceived hygiene, and is experienced through judgment, shame, discrimination, and social exclusion. The consequences of stigma extend beyond self-perception to influence social participation, mental wellbeing, and healthcare avoidance, creating self-perpetuating cycles of poor oral health and anticipated stigma. Strategies reported in the literature include seeking transformative dental treatment, adopting resilience-building and coping strategies, and promoting trauma-informed and non-judgemental dental care, although population-level interventions were notably absent. Collectively, these findings demonstrate that oral health stigma is a distinct and underexplored health-related stigma with significant implications for individuals and health systems, warranting further empirical attention.

## Supplementary Information

Supplementary Material 1

## Data Availability

All data used in this study exist is derived from published material available within the public domain and reference within the manuscript.
