# Supplementary material for: Unmasking oral health stigma: a qualitative scoping review
Source: BMC Oral Health. 2025 Dec 17;26:345. doi: 10.1186/s12903-025-07329-9 (PMC12922240; doi:10.1186/s12903-025-07329-9)
Supplement: Supplementary file 1 — Supplementary Material 1 [file 12903_2025_7329_MOESM1_ESM.docx]

**Supplemental material: Unmasking stigma about oral health: a qualitative scoping review**

*Supplemental Table 1: PPI across scoping review stages (Pollock et al. (2022) and Preston et al. (2023))*

| Scoping review stage* | Patient and public involvement | Means of involvement | Extent of patient and public involvement |
| --- | --- | --- | --- |
| Prior to conducting the scoping review | 1. Patients and the public will be invited to complete a short survey to share their lived experience of how oral health has impacted their life. 2. Patients and the public will be invited to decide which aspects of the scoping review they wish to be involved with and to what extent they wish to be involved (consulting, collaborating, directing). 3. Patients and the public will be invited to identify existing skills and to identify where training and guidance will be required at each stage of the scoping review. | 1. Survey 1: lived experience and perspectives of oral health stigma (used only to support search term development, not used as data for study)  2. Survey 2: skills, willingness to be involved and availability | Collaboration |
| Identifying the research question | PPI members will be invited to discuss the relevance and importance of the research question. They will also be invited to shape the research question and to advise whether key concepts such as stigma are appropriate and relevant. | Group discussion online | Collaboration |
| Identifying relevant studies | Through conversation with PPI and survey we identified additional terms for search including lay terms for oral health conditions, stigma and shame. | Group discussion online | Collaboration |
| Collating, summarising and reporting the results | 1. PPI members will be invited to participate in interpreting the findings from the included papers 2. PPI members will be invited to review themes for appropriateness and relevance 3. PPI will be invited to consider implications for research, policy and practice. | Review of the early interpretation stage of the findings and contribution to interpretation/ discussion points. | Collaboration |
| Consultation | 1. PPI will be invited to co-author the manuscript 2. PPI members will be invited to disseminate within their networks | Reviewed early and final manuscripts. | Collaboration |
| PPI were not involved in extracting data from the studies, but were involved in each of the other stages of the scoping review process either through consultation or collaboration. | | | |

Supplemental Table 2: Definitive search terms

| **Search terms** | **Database** | **Number of papers** | **Date of search** |
| --- | --- | --- | --- |
| •  Stigma* OR blame OR blaming OR discriminat(*) OR prejudice OR (social AND exclusion) OR ostracise OR disgust.  OR bullying OR teasing OR embarrass(*), OR shame OR ashamed OR shaming OR *employed OR intelligence OR self-esteem OR successful OR unsuccessful OR confidence OR confident OR happiness OR self-conscious OR insecure OR anxious OR neglect* OR disadvantage* OR debilitat*, OR criminal*  **AND**  Dentistry OR dental OR oral health OR teeth OR mouth OR (dental AND appearance) OR (teeth AND appearance) OR decay OR caries OR (rotten AND teeth) OR periodontal OR (gum AND disease) OR (missing AND teeth) OR denture* OR (access AND dental) OR dentist* OR (media AND dental) OR dentist* OR (television AND dental)  **AND**  Qualitative OR (focus AND groups) OR interviews OR observation OR (mixed AND methods) | **Web of Science** | **5940** | **16^th^ July 2024** |
|  | **Medline via ovid** | **3311** | **16^th^ July 2024** |
| **Total** |  | **9251** |  |


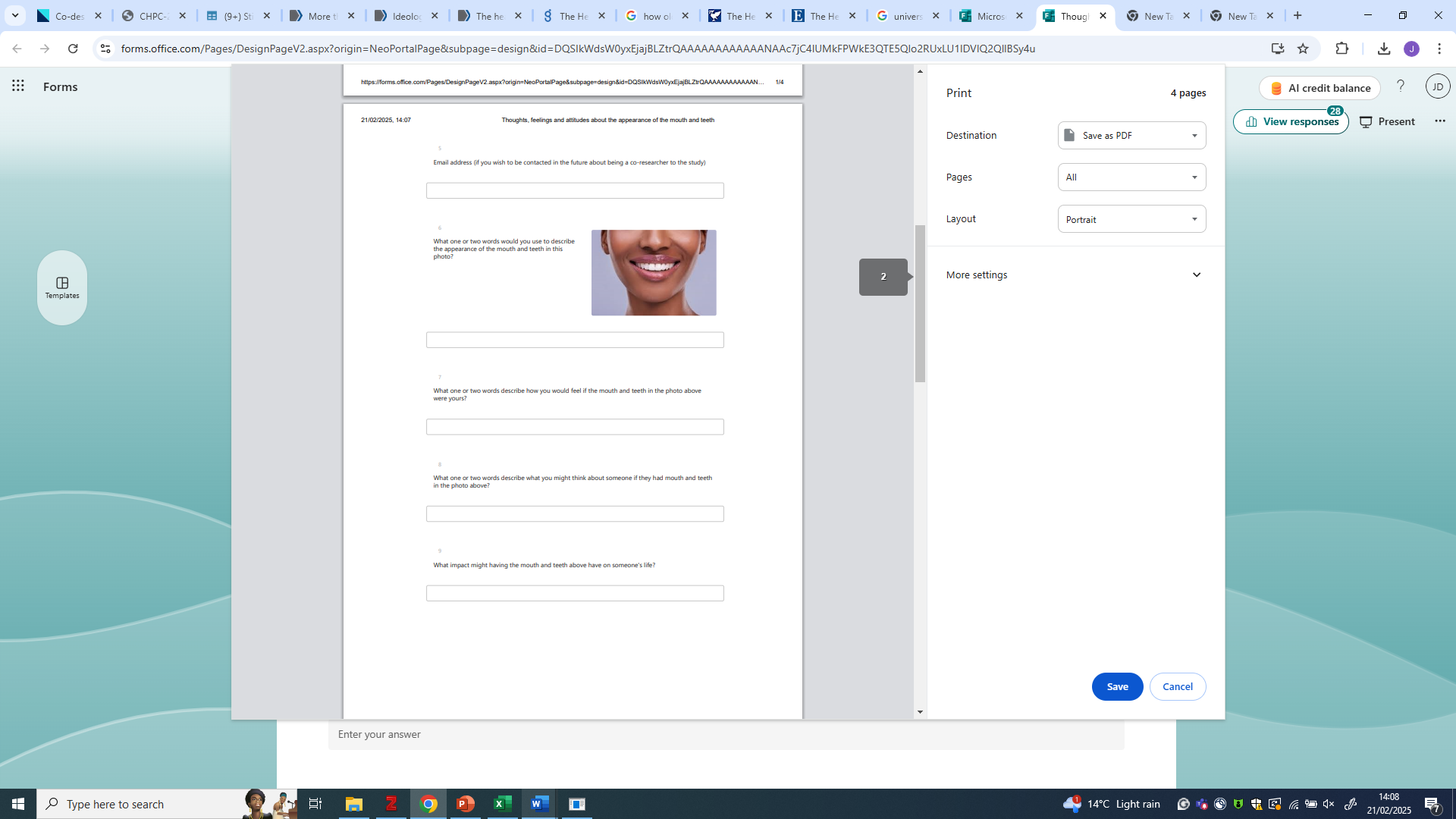

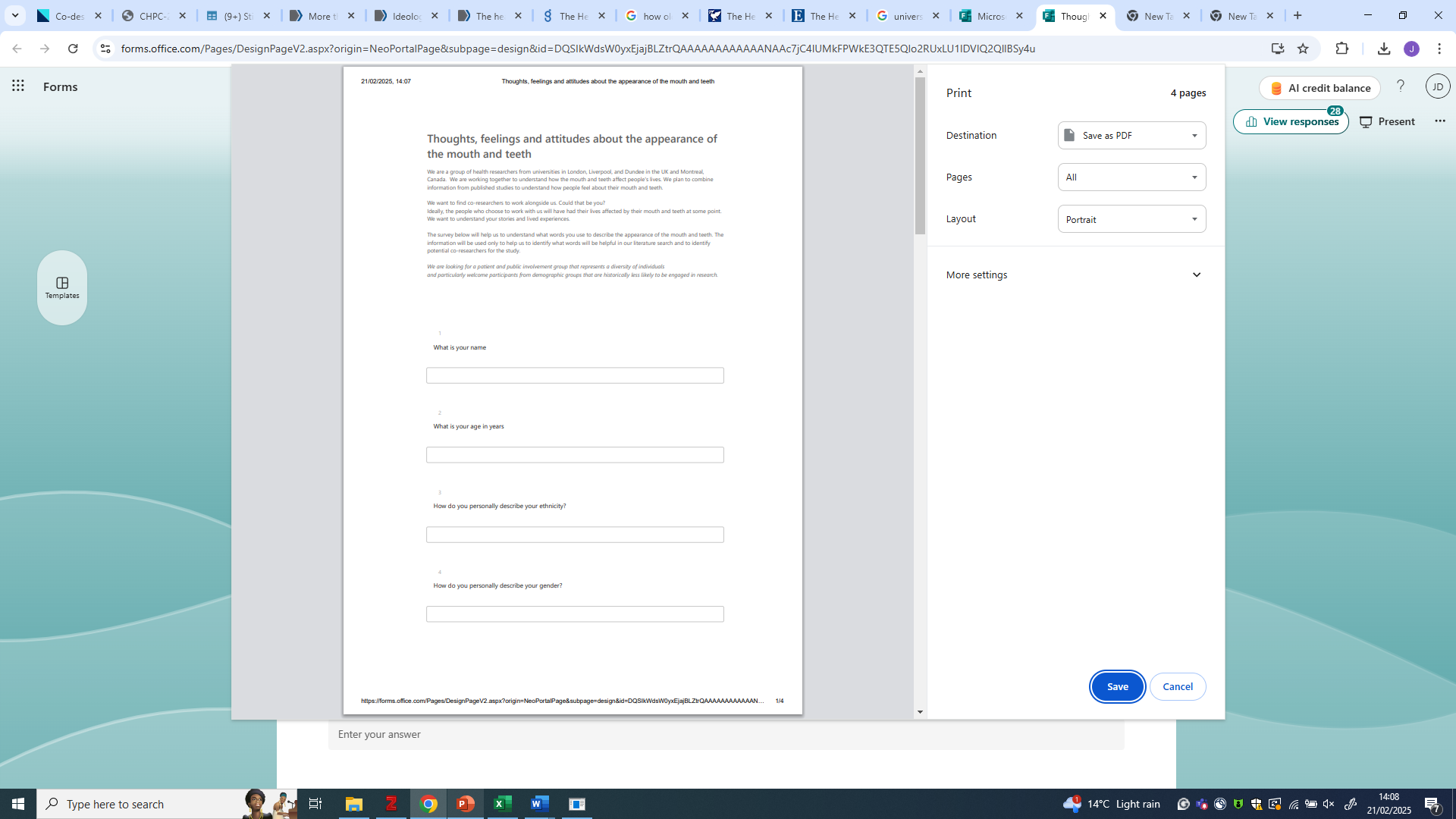


Figure 1: PPI survey of lived experiences and perspectives of oral health stigma


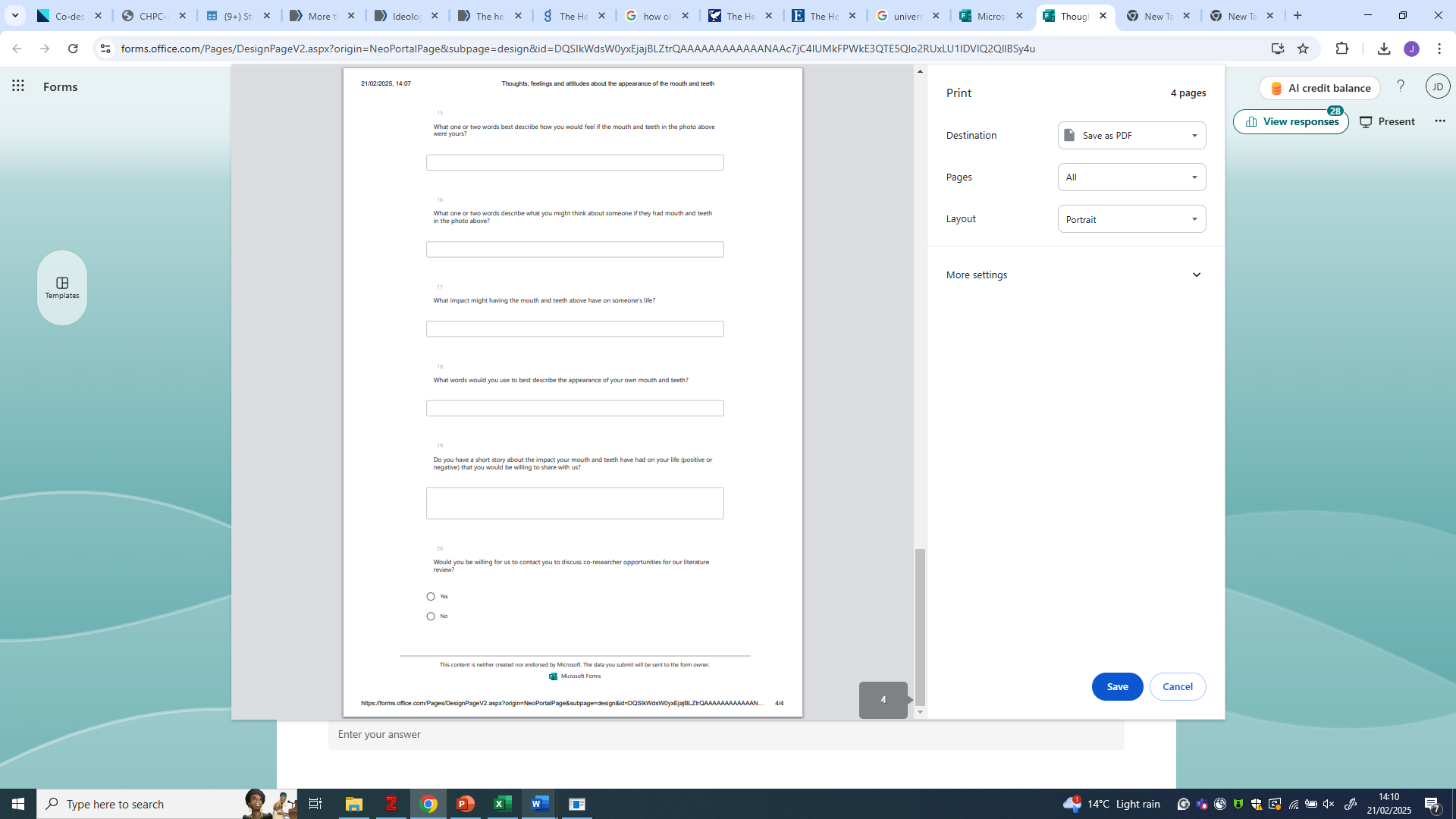

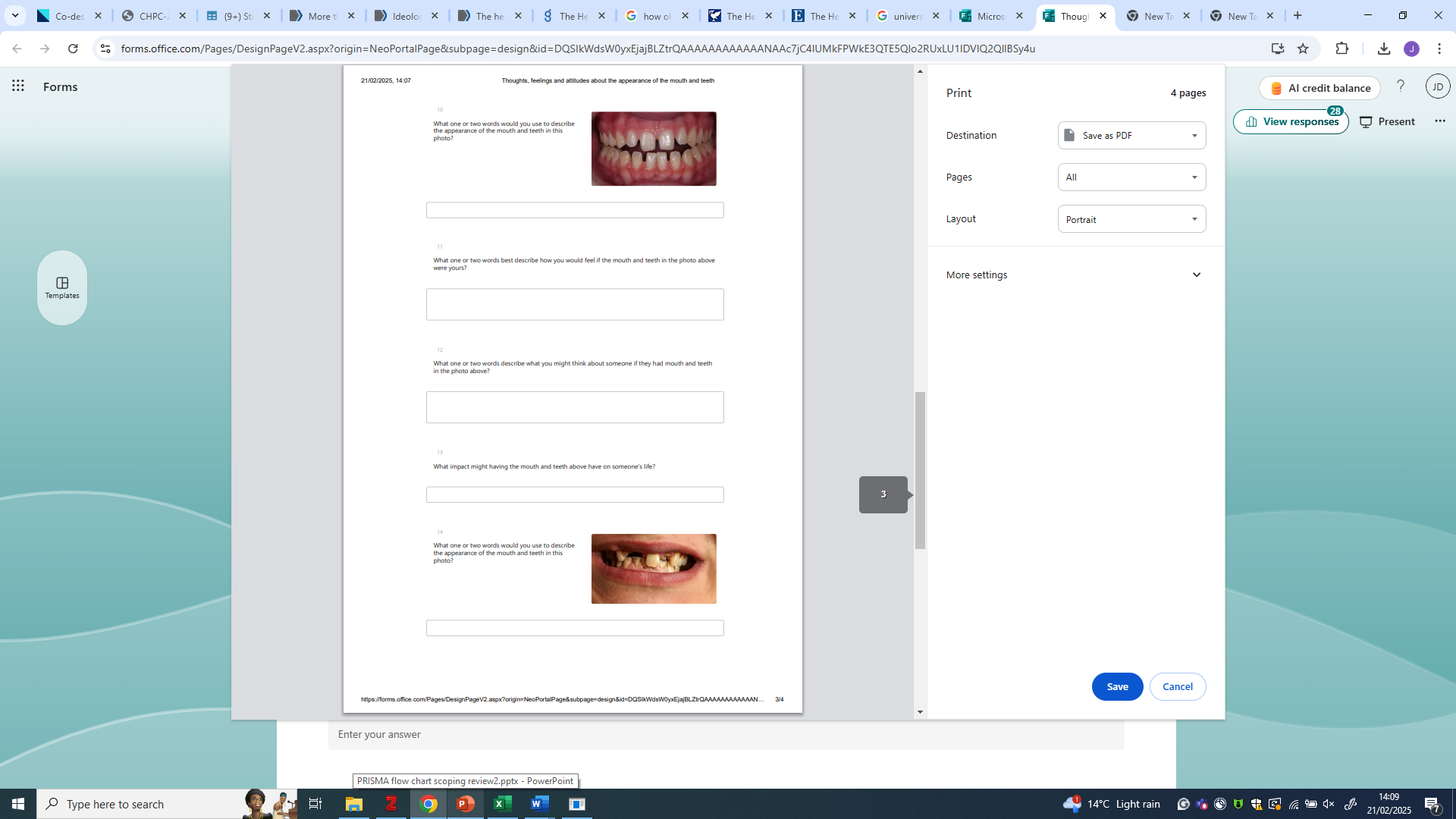


*Supplemental Table 3: Illustrative quotes for each theme and subtheme.*

| Meta theme | Subtheme | Extracted qualitative quotes | Study, Participant characteristics |
| --- | --- | --- | --- |
| Oral health stigma understood in physical terms related to attractiveness and disgust | NA | "In the first place, I would emphasise appearance, whether you like it or not, the thing that counts most in people's life is appearance, isn't it? ". | Folchini et al. (2023).  People who have had orthosurgical treatments. |
|  |  | “Your face changes because of your flabby mouth, that ugly, soiled mouth”; “we don’t look like anything at all. We lose our teeth; we lose everything…Being toothless is being poorly finished…a smile is very precious, isn’t it?” “I’m incomplete. Who is going to say that I am complete? Toothless like I am here”. “Look at me; you would feel this way, uneven, a different thing”. “You look ugly; you feel ashamed of even going out, talking to people”. | Paula et al. (2019).  People who have experienced tooth loss and denture rehabilitation. |
|  |  | “There’s no doubt that if someone is in this situation, besides your loved ones, many people hate you and find you disgusting. You even find yourself disgusting. For example, if you are eating in the company of other people, even if we’re not using the same container and each person has their own plate, some don’t like the fact that you’re sitting with them at the same table. “ | Kagone et al. (2022)  Person affected by NOMA. |
|  |  | “Maybe at a wedding or something, you know, where you’re put around a table. Ach well just embarrassment. You didn’t want it, you know yourself, it would turn people’s stomach.” | Semple et al.(2019)  Oral cancer survivor living with an obturatotor. |
|  |  | “Teeth are very important, it’s the first thing people notice when they look at you, I believe. This affects my social relationships, I believe it’s because of bad breath, and also because of the yellowed and crooked teeth” | Salvador et al. (2021)  Adult in need of endodontic treatment. |
| Oral health stigma: experienced as judgement, shame and exclusion |  | “I feel ashamed. Something’s wrong. Everyone around me has these beautiful teeth. I don’t, and something is wrong” ^1^  ”Shame was mentioned as a barrier to creating relationships with others, particularly during school years, due to low confidence or selfesteem, “I know how hard it was for me during school… I didn't interact with anyone because I was so self-conscious about my teeth, and I actually missed out on meeting a lot of really good people.”^2^ | Protudjer et al.(2022)  Older person with arthritis. |
|  |  | “Cause I’ve let me teeth go. Know what I mean? As I said to you, they’re like, my teeth are like an alehouse piano: one white, one black and one missing. You know, [like] the keys. Or a cemetery, d’you know what I mean? That’s my fault but I was like, forget today, you know what I mean. There’s no tomorrow.” | Van der Zande et al. (2021).  Adults without regular dentists who attended urgent dental care in England. |
|  |  | “It’s embarrassing… walking around with a kid, smiling, with teeth [rotten] like that. It’s like neglecting your kid” | Poirer et al. (2022)  Indigenous mothers. |
|  |  | “They [dentists] think that I wrecked my teeth and I shouldn’t have done that” | Brondani et al.(2017)  Residents from community treatment centers with a history of addiction and mental illness. |
|  |  | “Part of me was seeking to destroy something that I highly valued. It was part of the whole self-sabotage phase I was going through. Part of me also wanted something; a physical symbol of the inner turmoil that I was going through. I just wanted something physical to show for it. … Because I had nothing left that someone could point to that say that was real. External visuals validated it, and I wanted other people to look [at me] and say ‘that’s wrong, that’s bad’” | Ho et al. (2017)  Patients with mental illness in Australia. |
| Oral health stigma: self-consciousness that drives concealment |  | ”It’s funny because my boss said, 'You need to smile.' It's like, 'I'm trying.' He said, 'No, you're not. You're still putting your hand over your mouth. Put your hand down and smile.’ That takes a lot to get used to when you’re used to doing something for 15 years…I didn’t have a very pretty smile…” ^3^ | Gragoll et al.(2021)  Healthy Michigan Plan beneficiaries |
|  |  | “When my teeth were broken, I didn’t dare go, because I was ashamed to show up at the dentist’s looking like that” ^4^ | Gragoll et al.(2021)  Irregular dental attenders in Mecklenburg. |
|  |  | “I'm really conscious that I'm talking to them, you know . . . especially if they've got lovely teeth I'm thinking, what will they think of me?” | Standford et al.(2014)  Adult orthodontic patients |
|  |  | “At the beginning when I heard that Ihad cancer, the first moment Ithought this: I’ll hide, I will not tellanyone. Because I was ashamed…afraid of being rejected, ’cause people do not understand. And in my case,right in the face… then, well… I was afraid of being rejected in society.Thus, in the first moment I wanted to hide.” | Costa et al.(2014)  Patients with head and neck cancer in Brazil. |
| Strategies used to address oral health stigma | Recognising the transformational value of dental treatment | “My self-confidence really, it [dental treatment] will do wonders for that. There's nothing worse than talking to people in the street and talking to them keeping your mouth covered so they can't see the damage that has been done. And, as I say, it will help greatly with my confidence with the chances of getting work.” | Caton et al.(2016)  Homeless people accessing an outreach dental service. |
|  |  | “Yes. Yes. They’re not afraid anymore. When my teeth were very bad, I was not so friendly looking I guess, but now I’m in recovery. It’s a big difference.” | Kerr (2018)  Adults with limited access to dental care due to financial issues. |
|  |  | “To be honest, I’d like to be able to smile properly without wanting to hide all the time.” | Poirer et al. (2021)  Indigenous mothers |
|  |  | “I was really happy. After the operation, the very people who had been nasty to me were the first to come and wish me well. [ . . . ] From that moment on, I started to regain my pride, my joy of living, and this is increasing day by day.” | Kagone et al. (2022)  Person affected by NOMA |
|  | Adopting coping strategies to build resilience and foster social connections | "I discussed my feelings and dental problem with my friends and family, and they comforted me by understanding and listening to my feelings. My friend really helped me to overcome this and also rightly guided and motivated me to seek consultation and appropriate treatment with confidence."^5^ | Abideen et al. (2024)  People who experience oral health stigma |
|  |  | "I took an active role in advocating for the eradication of dental stigma in the community as well as advocating for equality and inclusion for people with oral problems. I also experienced stigma for a very long time, and the efforts and eagerness to put my oral health back on track through learning and awareness have given me the confidence to tell my story and influence positive change." | Abideen et al. (2024)  People who experience oral health stigma |
|  |  | “I did not like and to this day I do not like… looking in the mirror…But now I accept myself more…because… in the beginning it was not easy, right. A vain person…because I was vain… But today I’m calmer.” | Costa et al.(2014)  Patients with head and neck cancer in Brazil. |
|  | Promoting trauma-informed dental care to eliminate judgment | “They didn’t look grossed out when they saw how unclean my teeth were…you always worry about how they’re gonna react, when they look in your mouth and see the neglect…They didn’t treat me as if I was any lower than they were…I didn’t feel judged”. | Herlick et al.(2020)  Women who have been involved with the criminal justice system. |
